# A truncated aptamer-based electrochemical sensor for sensitive Ara h 1 determination on gold nanoparticle-modified screen-printed electrodes

**DOI:** 10.1007/s00604-026-08153-w

**Published:** 2026-06-05

**Authors:** Songül Kırlak Kara, Serdar Şanlı, Burhan Bora, Serkan Şen, Mutlu Sönmez Çelebi, Serap Evran

**Affiliations:** 1 Division of Chemistry, Institute of Science, Ordu University, Ordu, Turkey; 2 Department of Chemistry, Faculty of Science and Arts, Ordu University, Ordu, Turkey; 3 Department of Biochemistry, Faculty of Science, Ege University, Izmir, Turkey

**Keywords:** Food allergy, *Arachis hypogaea*, Aptamer, Biosensor, Electrochemical sensor, Mobile potentiostat

## Abstract

Food allergy, a worldwide health problem due to its rising prevalence, is an immune reaction to allergens. Among these, peanut allergy poses significant health risks and underscores the critical need for accurate detection methods. This study focuses on developing an electrochemical aptasensor based on a gold nanoparticle and Ara h 1 specific aptamer decorated screen-printed electrode for the rapid, sensitive, and portable detection of a major peanut allergen. Integrated with mobile devices, the aptasensor allows users to obtain experimental results effortlessly and conveniently. The developed electrochemical aptasensor operates by measuring changes in charge transfer on the electrode surface upon the selective binding of Ara h 1 to the immobilized aptamer. The aptasensor effectively identified Ara h 1 residues in food samples, exhibiting a linear detection range of 500–25,000 ng/mL and a detection limit of 500 ng/mL. The aptasensor’s applicability was verified using food samples with measurements performed via a mobile potentiostat.

**Graphical Abstract:**

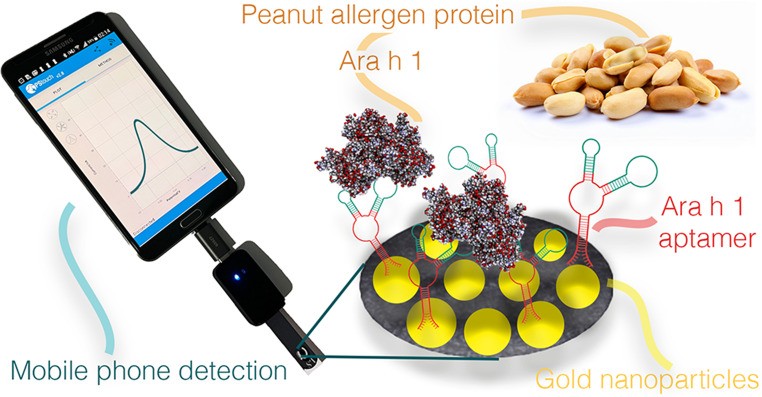

**Supplementary Information:**

The online version contains supplementary material available at 10.1007/s00604-026-08153-w.

## Introduction

Food allergy represents a significant global public health concern, with peanut allergy being one of the most severe due to its potential to trigger life-threatening anaphylactic reactions [1, 2]. Among the various peanut proteins, Ara h 1, a 65 kDa cupin-family vicilin, is recognized as a major heat-stable allergen that maintains its immunoreactivity even after thermal processing [3–6]. To protect allergic consumers, regulatory frameworks such as the VITAL 3.0 (Voluntary Incidental Trace Allergen Labelling) have established reference doses and action levels for industrial food production. Consequently, there is an urgent demand for analytical tools capable of rapid and reliable allergen monitoring in complex food matrices [7–9].

Currently, Enzyme-Linked Immunosorbent Assay (ELISA) remains the gold standard for allergen detection due to its high sensitivity and specificity [10]. However, ELISA is predominantly a laboratory-based technique requiring long incubation times (2–4 h), specialized equipment, and trained personnel, making it unsuitable for real-time on-site screening. While Lateral Flow Devices (LFDs) offer portability, they often lack quantitative precision and are prone to false-negative results at high allergen concentrations. In this context, electrochemical aptasensors have emerged as a superior alternative, combining the high affinity of aptamers with the portability, low cost, and rapid response times of electrochemical platforms [11, 12].

Aptamers, often referred to as “chemical antibodies,” offer distinct advantages over traditional antibodies, including superior thermal stability, ease of chemical modification, and cost-effective synthesis [13–15]. Since the first report of an Ara h 1-specific aptamer by Tran et al. in 2013, several electrochemical platforms have been developed [16, 17]. Most of these sensors utilize the original 80-mer full-length aptamer sequence. However, the use of long DNA sequences can lead to high steric hindrance, reduced packing density on the electrode surface, and increased synthesis costs, which may limit the performance and industrial feasibility of the resulting biosensor [18, 19].

Aptamers’ specificity as target recognition elements makes aptasensors ideal for selectively detecting targets in complex matrices like food, blood, or pathogens [20]. Aptasensors can be categorized based on their transduction mechanisms, which include electrochemical, optical, and field-effect transistor-based methods [21]. Aptamer-based electrochemical biosensors have emerged as a significant technology for the rapid, sensitive, and selective detection of biomolecules. Aptamer technology is able to advance the development of nucleic acid biosensors for a broad spectrum of analytes due to the distinctive 3D structure of single-stranded DNA/RNA. Aptasensors have been the subject of in-depth investigation in a wide range of areas, including clinical diagnostics [22–24], food safety [25–30], food allergen detection [31–33], environmental monitoring [34], protein detection [35, 36], virus detection [37–40], small molecule detection [41–43], and cell detection [44].

While gold nanoparticles (AuNPs) are widely recognized as excellent transducer elements due to their high surface-to-volume ratio and superior conductivity, their role in this study goes beyond simple signal enhancement. The primary improvement lies in the synergistic combination of electrodeposited AuNPs with a truncated aptamer sequence. Unlike conventional full-length aptamers, the shorter sequence used in this work benefit from the nanostructured AuNP surface to achieve a more organized and dense self-assembled monolayer (SAM). The AuNPs provide a biocompatible, high-curvature platform that minimizes steric hindrance between the truncated strands, effectively increasing the number of active binding sites per unit area. This strategic integration not only facilitates faster electron transfer kinetics—evidenced by the significant reduction in R_ct_—but also stabilizes the bio-interface for robust performance in complex food matrices, a critical improvement for field-deployable ‘mobile’ sensing applications.

In this work, we present a novel, smartphone-integrated electrochemical aptasensor designed for the rapid industrial screening of Ara h 1. The core novelty of our platform lies in the strategic use of a truncated Ara h 1 aptamer. By removing non-essential flanking sequences and focusing on the core-binding region, we achieved a higher molecular packing density on a gold nanoparticle-modified screen-printed carbon electrode (AuNP-SPE). This structural refinement not only enhances electron transfer kinetics but also improves the sensor’s robustness against matrix interference. We utilized in-house produced recombinant Ara h 1, Cor a 11, and Ana o 1. This allowed for a rigorous validation of the sensor’s selectivity against high-purity structural homologs, ensuring the reliability of the bio-recognition event.

To bridge the gap between laboratory research and field applications, the developed aptasensor was integrated with a commercially available, smartphone-controlled portable potentiostat. This system provides a “lab-in-a-palm” solution capable of delivering quantitative results within 30 min. We demonstrate that the platform achieves a limit of detection (LOD) 500 ng/mL (0.5 ppm) in buffer, directly aligning with the sensitivity of commercial ELISA kits while offering the speed and portability required for industrial cleaning validation and raw material screening. The practical utility of the sensor was further validated through recovery studies in complex matrices, including chocolate and instant soup, confirming its potential as a robust tool for modern food safety management.

## Experimental section

### Materials and reagents

#### Materials

HAuCl_4_, K_3_Fe(CN)_6_, tris(2-carboxyethyl) phosphine (TCEP) and other reagents were supplied from Sigma Chem. Co. (St. Louis, MO, USA). All were analytical grade. 100 mM TCEP solution was prepared in ultrapure water and aliquots were stored at −20℃. 5’-Thiol modified Ara h 1 aptamer synthesized by ELLA Biotech (Fürstenfeldbruck, Germany) and HPLC purified DNA was delivered in lyophilised form. DNA was dissolved in ultra-pure water to give 100 µM final concentration. Aliquots of aptamer solution were stored at −20 °C. The Ara h 1 specific DNA aptamer used in this study is the truncated version of aptamer that was developed by Tran et al. [16]. The sequence of DNA aptamer used in this study is, 5’-SH-TTTTTTGGGGGGGTCGAGCTGAGTGGATGCGAATCTGTGGGTGGGC.

### Instrumentation

PalmSens 4 and Palmsens Sensit Smart potentiostats (Palm Instruments, Houten, Netherlands) driven by PSTrace 5.9 software on PC and PStouch on mobile phone respectively were used for differential pulse voltammetry (DPV), cyclic voltammetry (CV) and electrochemical impedance spectroscopy (EIS) experiments. PalmSens 4 was used for optimization experiments and, Sensit Smart potentiostat was specifically utilized for the final real sample experiments.

Electrochemical measurements were carried out on SPEs. The polyvinyl chloride (PVC) screen-printed three-electrode (SPE) system contains Ag/AgCl as reference and two carbon electrodes as a 3 mm working electrode and counter electrode. SPEs were purchased from Life Sense Teknoloji San.Tic.Ltd.Şti. (Samsun, Türkiye) and used for all electrochemical measurements. Bare SPEs were pre-treated by five scans of cyclic voltammetry using 0.05 M HCl solution before the experiments in order to eliminate impurities. Graphpad version 5.03 was used for graphics and statistical analysis.

Electrochemical measurements were performed using a commercially available, smartphone-controlled potentiostat (Sensit Smart, PalmSens, Netherlands), which was selected for its high precision and portability, facilitating the transition from laboratory validation to field-based food safety monitoring.

### Preparation of the SPE/AuNP platform

Two different surface modification strategies were explored through preliminary experiments. The first method utilized synthesized gold nanoparticles applied to the electrode surface using the drop-casting method. 100 mL of %0.001 HAuCl_4_ solution was stirred until boiling. Then 3 mL of %1 sodium citrate solution was added to the solution and observed for color change. A reddish color indicates well-prepared gold nanoparticles. The synthesized nanoparticles were drop-cast onto the electrode surface in a volume of 5 µL.

Conversely, the second strategy employed potentiostatic deposition to achieve surface modification. SPE working electrodes were coated with AuNP by the electrodeposition method at a constant negative potential of −0.4 V with 2 mM, 75 µL HAuCl_4_ solution for 25 s. After AuNP decoration, the electrodes were carefully rinsed with pure water and dried with nitrogen gas and then characterized by using CV and EIS and SEM techniques.

**Scheme 1:**
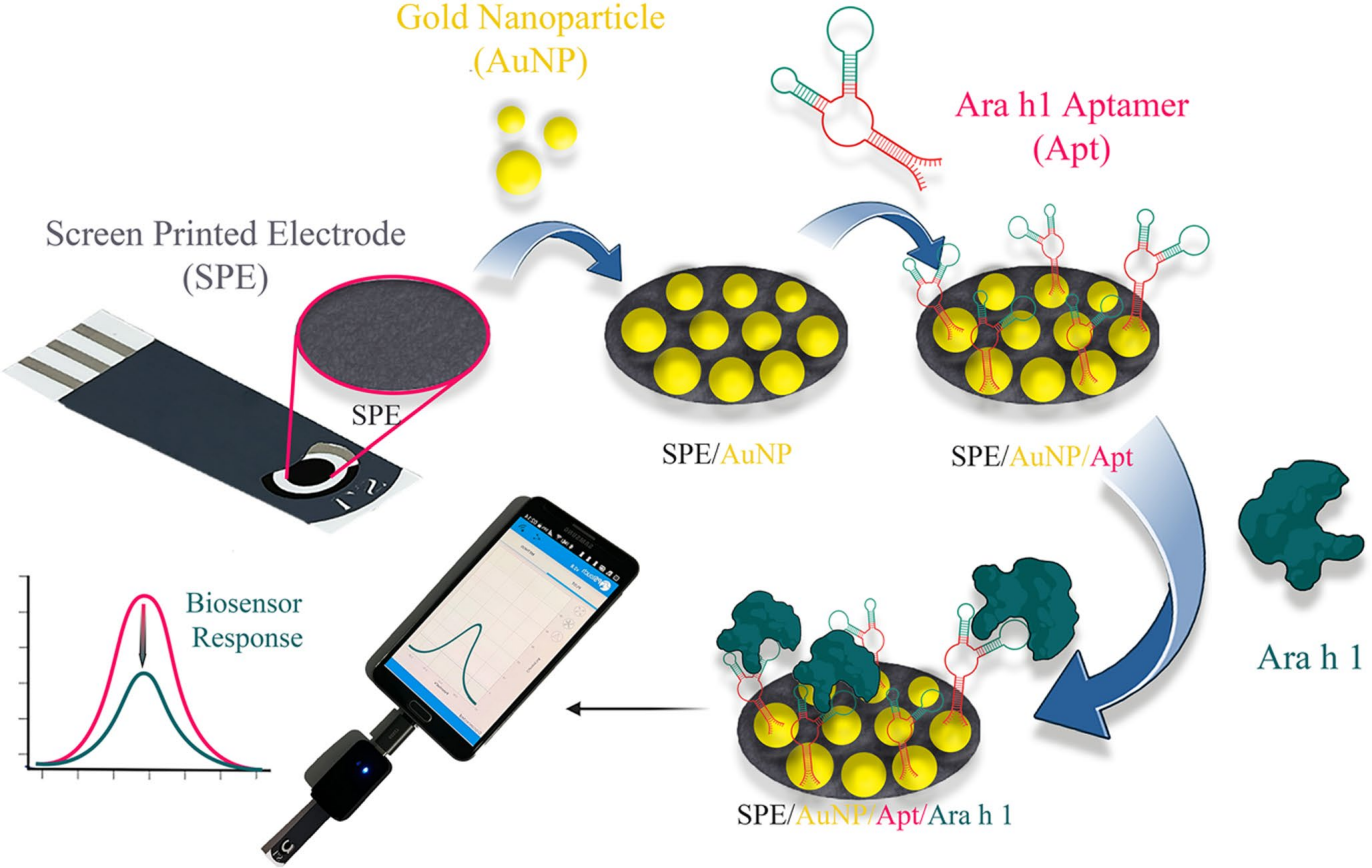
Design of SPE/AuNP/Apt sensor

Stock aptamer solution was diluted to various concentrations from 0.01 µM to 2 µM in binding buffer (PBS supplemented with 2 mM MgCl_2_ and %0.05 Tween-20). 2 mM TCEP was included in aptamer solution in order to reduce 5’-thiol groups. TCEP introduced aptamer solution was immediately transferred to thermal block that was preheated to 95 °C. After incubating the aptamer solution at 95 °C, it was left to cool on a bench for 30 min to reach room temperature. 5 µL of properly folded and thiol reduced aptamer solution was then applied to the surface of a gold nanoparticle-coated screen-printed electrode (SPE/AuNP). Surface immobilization of aptamers was achieved by establishing coordination bonds between the thiol group on the aptamers and gold. Following a 1-hour incubation period, electrodes were subjected to three washes with PBS-T (Binding/washing buffer; PBS-T: 0.024 gr KH_2_PO_4_; 0.18 gr Na_2_HPO_4_.2H_2_O; 0.02 gr KCl; 0.019 gr MgCl_2_; 0.05 gr Tween-20; V = 100 mL with ddH_2_O. The pH should be in the range of 7.35–7.45) to remove non-bound aptamers. The resulting biosensor was denoted as SPE/AuNP/Apt (Scheme 1).

### Aptasensor fabrication

Recombinant allergen proteins were expressed in *E. coli*. Codon optimized gene sequences for Ara h 1 and Cor a 11 were synthesized and cloned into pUC-GW-Kan vector by Genewiz (NJ, USA) and delivered to us in lyophilised form (for sequences of allergen proteins fig S2). Codon optimized gene sequence encoding Ana o I was synthesized and cloned into pIDT-Kan by IDT and delivered to us in lyophilised form. Protein encoding gene regions cloned to pET-28a protein expression vector from the restriction enzyme recognition sites *NdeI* and *XhoI*. Gene-cloned expression vector was transformed to *E. coli* T7 Express I^q^ (NEB, C3016) chemical competent cells and single colonies were selected for protein expression. After inducing protein expression by adding 300 µM IPTG to the bacterial culture, the culture was incubated at 18 °C for 36 h. Bacteria were separated by centrifugation and homogenized by ultrasonification. The resulting supernatant was then obtained by centrifugation to isolate the soluble recombinant protein. Clear lysate was loaded to Cytiva HisTrap FF Crude columns that connected to ÄKTA FPLC system and purest fractions collected after gradient elution with 500 mM imidazole. Purity of recombinant allergens was evidenced by %12 SDS-PAGE analysis [45]. Reactivity of Ara h 1 against immobilized antibodies was tested by using commercial R&D systems peanut allergen sandwich ELISA kit. Bradford protein assay was utilized to determine protein concentration [46].

### Electrochemical measurements and real sample preparation

Upon incubating the Ara h 1 protein sample on the electrode surface, the SPE/AuNP/Apt sensor undergoes surface coverage by the proteins, causing a change in the charge transfer across the surface. To monitor this change in charge transfer, 75 µL of a 5 mM [Fe(CN)_6_]^(3−/4−)^ in 0.1 M KCl solution (HCF redox mediator) was dropped onto the surface both before and after the addition of proteins, and the biosensor response was examined using the differential pulse voltammetry (DPV) technique within the voltage range of −0.4 V to + 0.6 V (E step: 0.0025 V, E pulse: 0.035 V, t pulse: 0.07 s, Scan rate: 0.017 V/s). The difference between the initial peak current (I₀) and the peak current obtained after protein binding to the surface (SPE/AuNP/Apt/Ara h 1), denoted as I, was accepted as the biosensor response (∆µA).

1 $$\triangle\mu A=I_0-I$$

Prior to all measurements, the surface was washed three times with PBS-T, followed by one wash with HCF. To determine the optimal binding time of Ara h 1 on the surface, the interaction between SPE/AuNP/Apt and Ara h 1 was allowed to proceed for a duration ranging from 10 to 75 min before washing steps. Following the identification of the optimal interaction time, the analytical characterization of the aptasensor was conducted by testing Ara h 1 proteins on the SPE/AuNP/Apt at concentrations ranging from 500 to 50,000 ng/mL. All fundamental optimization experiments (including AuNP deposition, aptamer immobilization times, and concentration studies) as well as the primary calibration curve were performed using the PalmSens 4.

In order to observe selectivity, cashew, hazelnut, and bovine serum albumin and Ara h 1 proteins at the same concentrations were tested on the designed SPE/AuNP/Apt surface. For repeatability and reproducibility trials, 25,000 ng/mL Ara h 1 was retested with three different SPE units. Standard deviation (± SD) and coefficient of variation (C.V.) were calculated with the results obtained from the experiments, and statistical analyses were conducted using the Anova test within the Graphpad software.

Instant soup, potato chips, and a chocolate-coated caramel biscuit (C.C. biscuit) were used as real samples. Real samples were selected from food items that did not include allergen warnings for peanuts. One gram of each food sample was fragmented and added to 25 mL of 50 mM PBS-T pH 7.4 buffer. Samples were shaken in the water bath at 65 ℃ for 30 min. 30 min of centrifugation at 15,000 rpm was performed to separate the layers. The aqueous phase remaining between the upper-fat layer and the lower pellet was carefully removed with the help of a micropipette. After filtration with a 0.45 μm syringe filter, samples were diluted 10-fold in PBS-T buffer. 5000 ng/mL Ara h 1 was spiked to prepared real samples and analyzed using SPE/AuNP/Apt aptasensor. The PalmSens Sensit Smart mobile potentiostat, integrated with a smartphone via the PStouch app, was specifically utilized for the final real sample experiments. PalmSense Sensit smart mobile phone potentiostat was used for real sample experiments. Biosensor’s recovery percentages of real sample results were calculated.

## Results and discussion

In comparison to conventional protein structured antibodies, aptamers possess several beneficial properties, including small size, structural flexibility, and excellent biocompatibility [20, 21, 34, 47–49]. In this work, an Ara h 1 sensor was developed by leveraging the superior properties of aptamers. The characterization and optimization results of the aptasensor demonstrated its high potential for detecting allergenic substances at field applications.

The core novelty of this work resides in the synergistic integration of a rationally truncated Ara h 1 aptamer with an electrodeposited AuNP-SPE interface. While the original 80-mer aptamer reported by Tran et al. [16] provides high affinity, its large molecular footprint limits the packing density on nanostructured surfaces. By removing the non-essential 20-nt primer-binding flanking sequences, we reduced the aptamer length by 50%. This truncation offers three distinct advantages: (i) Increased Packing Density: The smaller footprint allows for a higher number of active binding sites per unit area compared to the full-length sequence. (ii) Enhanced Electron Transfer: The shorter DNA chain facilitates faster charge transfer kinetics between the redox probe and the electrode, improving the signal-to-noise ratio. (iii) Reduced Steric Hindrance: Shortened sequences are more flexible and less prone to non-specific entanglement, which is crucial for maintaining selectivity in complex matrices like chocolate and soup.

While a slight increase in the dissociation constant (Kd) is often a trade-off in solution-phase truncation, our electrochemical results indicate that the increased surface density more than compensates for this, leading to a robust sensor that meets industrial ppm-level requirements.

### Recombinant allergen protein production

Genes encoding allergen proteins were successfully cloned to pET-28a expression vector. Sequences of allergen encoding genes verified by Sanger sequencing of isolated plasmid DNAs. Sequence verified plasmid DNA was transformed to expression strain *E. coli* T7 Express Iq. After heterologous expression of allergen proteins, IMAC purification was done and purest fractions of gradient elution were combined (Chromatogram of gradient elution and SDS-PAGE analysis results Supporting fig S3). SDS-PAGE analysis showed clear bands for the Ara h 1, Cor a 11, and Ana o 1 recombinant proteins at approximately 55, 45, and 25 kDa, respectively, indicating high purity (Supporting info Fig S4). Recombinant Ara h 1 was tested with Ridascreen peanut allergen ELISA kit. Ara h 1 exhibited high reactivity against immobilized antibodies supplied with the kit (Supporting info fig S5). Recombinant Ara h 1 and Cor a 11 were tested for their thermostability by incubating protein solution at 95 °C for 30 min. After incubation, centrifugation at 21,000 g was done before analyzing pellet and supernatant by using Bradford protein assay and no aggregation was observed, indicating correct 3D folding of recombinant proteins. Protein samples were frozen until use.

### Optimizations of aptasensor

The first step in developing the surface of the aptasensor is the deposition of AuNPs on the SPE surface. In the second step, aptamers were immobilized on these gold nanoparticles to obtain a selective surface.

Preliminary trials were conducted using two distinct methods for the AuNP-based surface modification. The first one involved drop-casting the synthesized citrate-stabilized AuNPs onto the electrode surface. The synthesized AuNPs were also characterized using UV-Vis spectrophotometry and FTIR. The characterization data have been included in the Supporting Information (Figures S6 and S7).

In the second approach, 75 µL of 2 mM HAuCl_4_ was dropped onto the SPE surface, and AuNPs were obtained using the chronoamperometry. Although similar results were obtained with both methods, surfaces obtained through the chronoamperometric method were observed to be more stable in terms of repeatability.

**Fig. 1:**
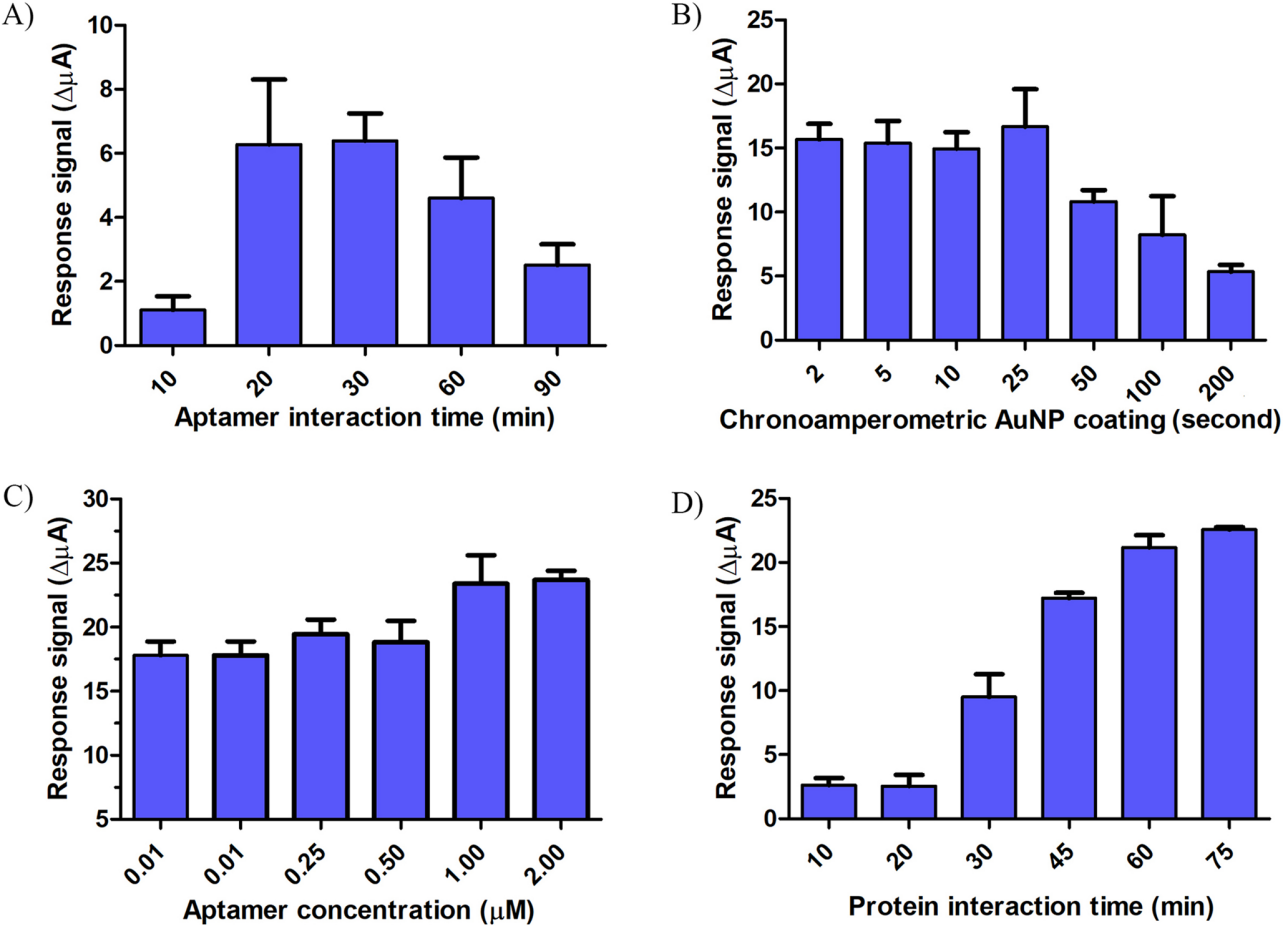
Optimizations of aptamer interaction time (**A**), AuNP electrodeposition (**B**), aptamer concentration (**C**), and protein interaction time (**D**)

The first optimization stage was aptamer interaction time on SPE/AuNP surface. 30 min of incubation time was the best result for aptamer incubation. As illustrated in Fig. 1A, the peak current reached its maximum at an incubation time of 30 min, followed by a discernible decrease at 60 and 90 min. This trend is attributed to the over-saturation of the electrode surface with thiolated aptamers. During the initial 30 min, a functional self-assembled monolayer (SAM) is formed, providing optimal spacing for target binding. However, prolonged incubation leads to an excessively high packing density, which induces significant steric hindrance. This congestion limits the conformational flexibility of the aptamer strands, hindering their ability to effectively undergo the structural changes necessary to bind the bulky Ara h 1 protein. Additionally, the accumulation of the negatively charged phosphate backbone of the DNA aptamers increases the interfacial electrostatic repulsion against the [Fe(CN)_6_]^3−/4−^ redox probe, thereby impeding electron transfer and resulting in a decreased current signal.

Chronoamperometric AuNP deposition was explored over varying durations ranging from 2 s to 200 s (Fig. 1B). A decrease in sensor response was observed after 25 s. It was observed that the excess amount and size of accumulated AuNPs on the surface increased conductivity, facilitated charge transfers, and led to a decrease in biosensor response. In the subsequent parts of the experiment, chronoamperometric AuNP coating was performed for 25 s.

To determine the optimum mole number of aptamers to be captured onto the surface, aptamers within the concentration range of 0.1 µM to 2 µM were tested on the aptasensor surface (Fig. 1C). The objective of the optimization step was to achieve a high sensor response while using as minimal aptamer quantity as possible for cost-effectiveness. Results obtained with 1 µM aptamer were comparable to those obtained with higher concentrations and were therefore deemed sufficient. Subsequently, the duration required for the interaction between Ara h 1 and the fabricated SPE/AuNP/Apt surface was investigated (Fig. 1D). It was determined that the SPE/AuNP/Apt surface became saturated with protein after 60 min. Calibration experiments involved an Ara h 1 incubation period of 1 h.

### Characterizations of aptasensor

**Fig. 2:**
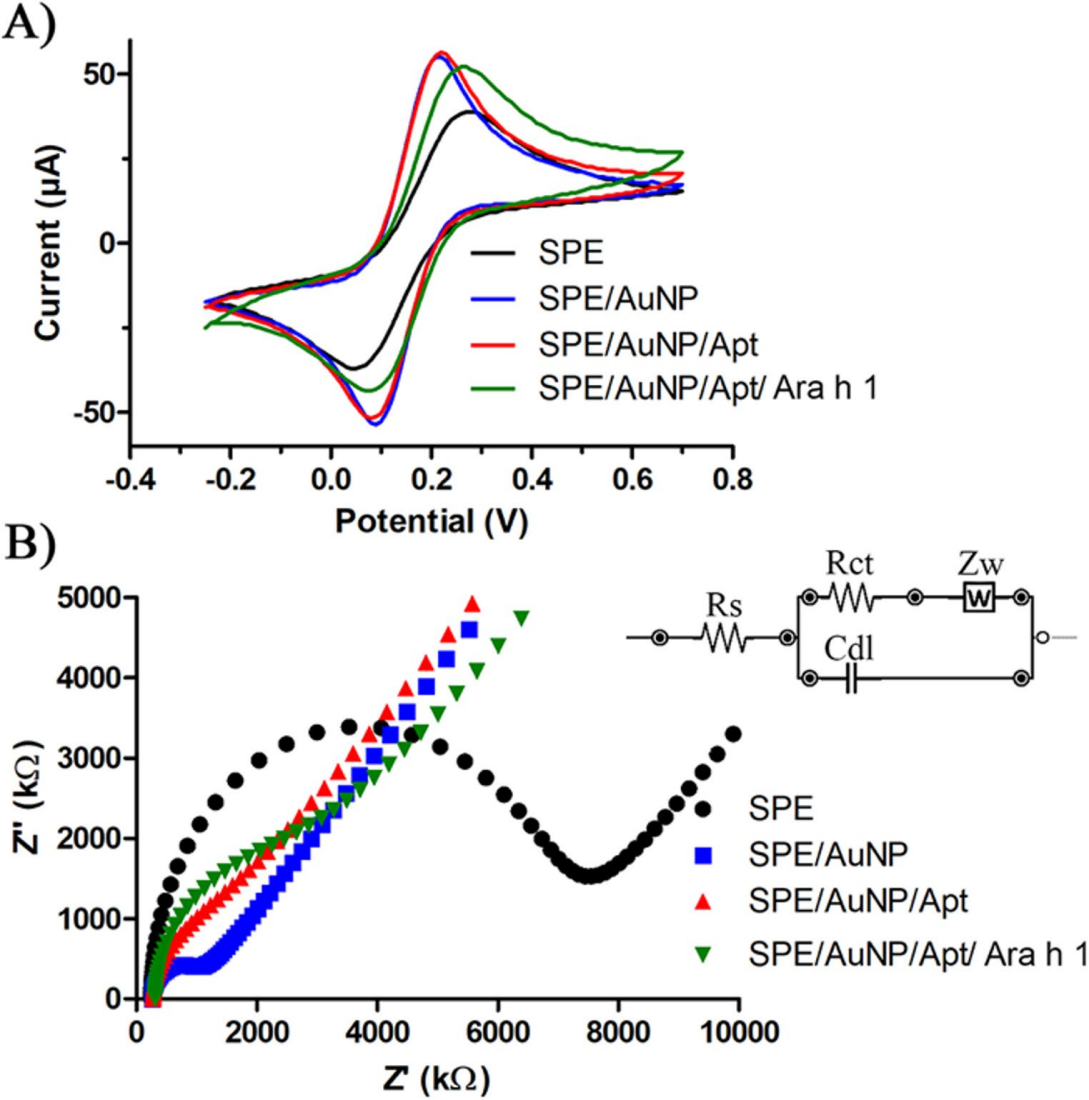
CV (**A**) and EIS (**B**) graphs of the construction steps of the aptasensor

The electrochemical characterization of the SPE/AuNP/Apt under optimal conditions was examined using CV and EIS techniques (Fig. 2). Cyclic voltammetry revealed that coating the surface with AuNPs resulted in an elevated charge transfer, which significantly amplified both anodic and cathodic peak currents and reduced peak-to-peak separation. The peak-to-peak separation, anodic and cathodic peak currents for all SPE/AuNP/Apt development steps are summarized in Table S2. Coordination bonds were formed between the thiol groups on the aptamers and AuNPs, facilitating the attachment of aptamers to the sensor surface. This binding resulted in a slight decrease in CV signals. The attachment of Ara h 1 molecules to the surface hindered charge transfer between the redox mediator and SPE/AuNP/Apt, leading to a noticeable reduction in CV peak potentials. The anodic peak current was recorded as 40.76 µA for bare SPE. It reached 62.41 µA for SPE/AuNP and 60.81 µA for SPE/AuNP/Apt, while SPE/AuNP/Apt/Ara h 1 decreased the peak potential to 49.11 µA. Conversely, cathodic peak currents were observed as −40.55 µA for SPE, −62.29 µA for SPE/AuNP, −58.13 µA for SPE/AuNP/Apt, and − 46.38 µA for SPE/AuNP/Apt/Ara h 1. Peak-to-peak separations were noted as 0.22 V for SPE, 0.12 V for SPE/AuNP, 0.14 V for SPE/AuNP/Apt, and 0.18 V for SPE/AuNP/Apt/Ara h 1.

The EIS results were found to be consistent with the CV findings, providing supportive evidence. To annotate the EIS measurements, Randle’s equivalent circuit was designed including Rs (solution resistance), Zw (Warburg impedance), Cdl (double layer capacitance), and Rct (change in electron transfer resistance) as shown in Fig. 2B inset circuit. Semi-circular peaks at higher frequencies at Fig. 2B indicate the Rct results of each modification steps. As expected, the Rct values decreased from 6502 Ω (SPE) to 700 Ω (SPE/AuNP) and 1067 Ω (SPE/AuNP/Apt) with AuNP and Apt modifications and increased to 2255 Ω (SPE/AuNP/Apt/Ara h 1) with Ara h 1 addition. The results of both CV and EIS techniques were indicated successful surface modifications of the SPE/AuNP/Apt/Ara h 1. Rs, Rct, Zw and Cdl measurements for all SPE/AuNP/Apt development steps are summarized in Table S3.

**Fig. 3:**
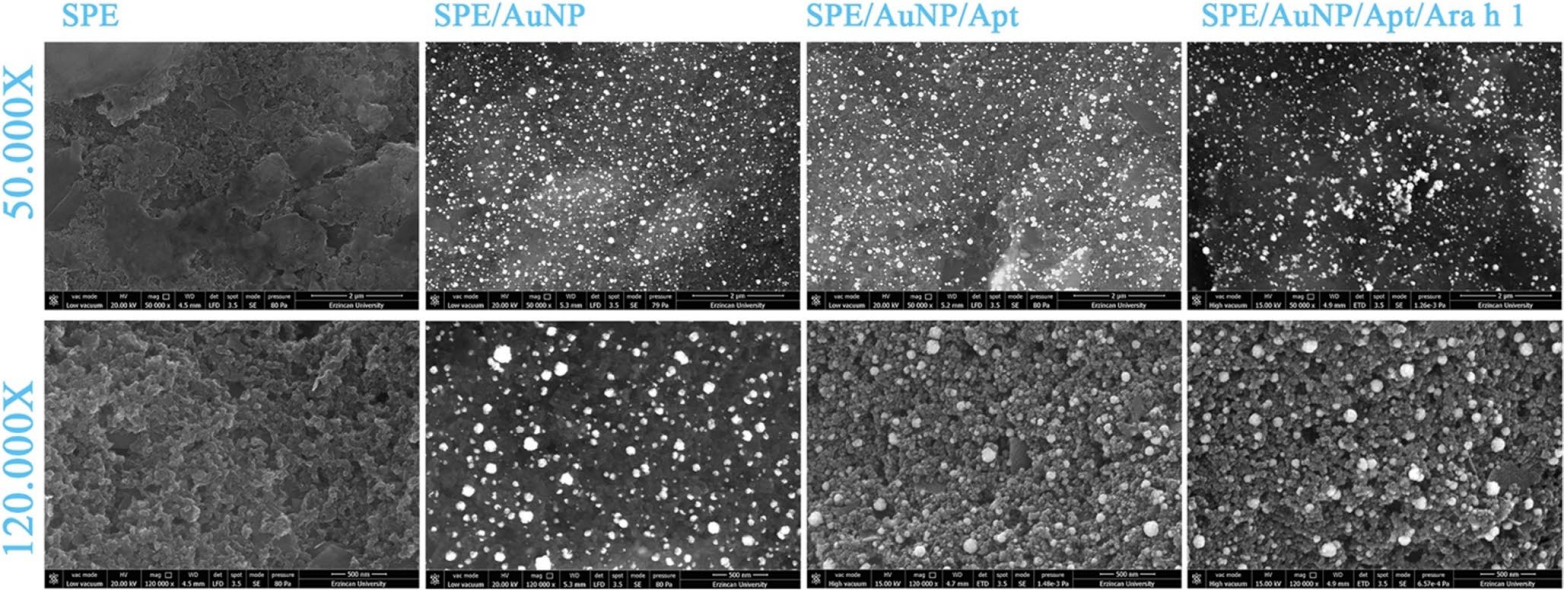
SEM images of the construction steps of the aptasensor

**Fig. 4:**
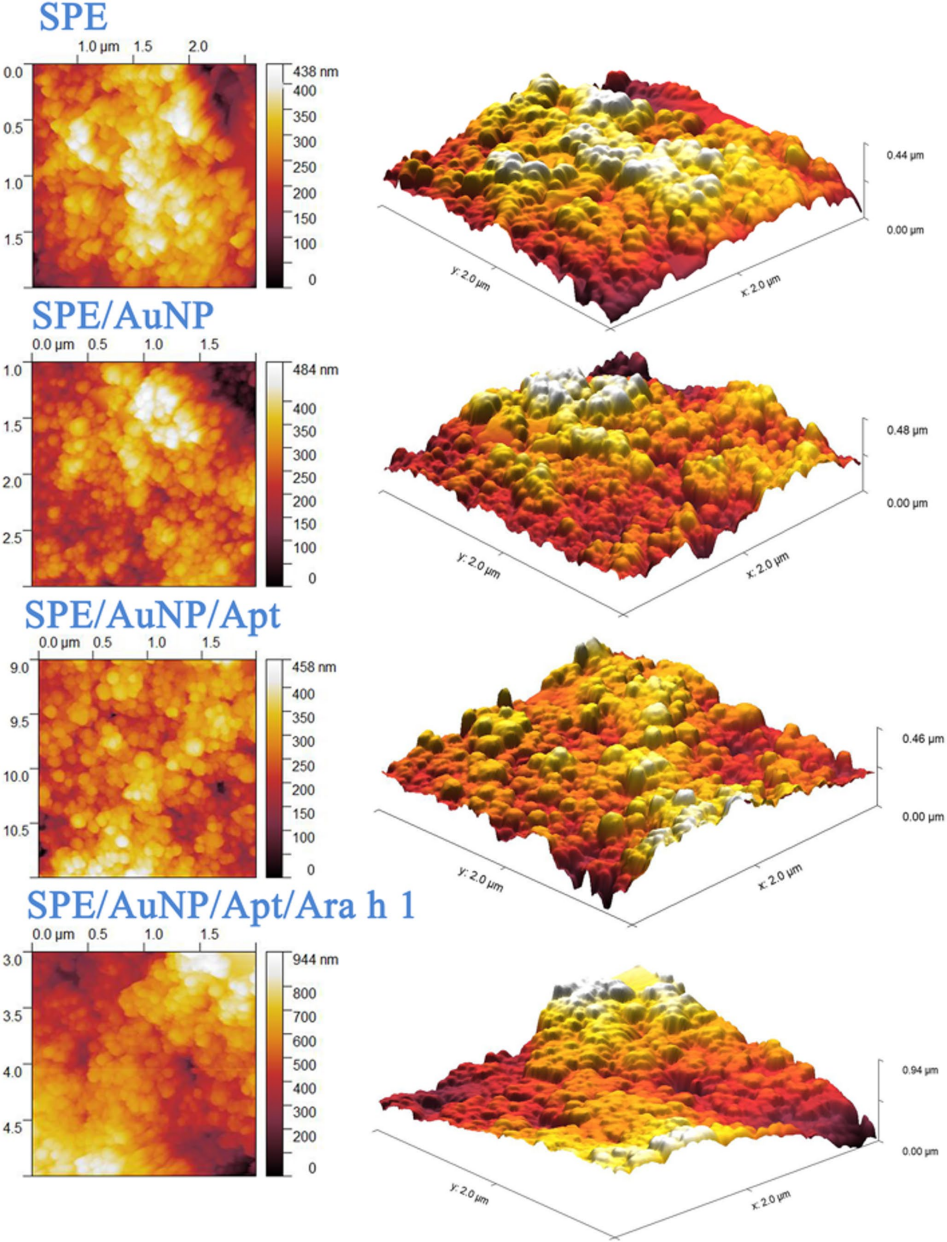
2D and 3D AFM images of the construction step of the SPE/AuNP/Apt/Ara h 1 sensor

SEM and AFM techniques were employed to characterize the surface topography. As shown in Fig. 3, AuNPs were uniformly distributed on the SPE electrode. Upon analyzing the SEM images, the addition of aptamers resulted in a more textured surface morphology. Although the Ara h 1 structure, with a molecular weight of 55 kDa [49], was not distinctly visible in the SEM images, it caused a noticeable change in surface roughness as measured by AFM. Surface roughness values (Sa, mean roughness) were found 58.91 nm for SPE, 61.04 for SPE/AuNP, 45.81 nm for SPE/AuNP/Apt and 127.72 nm for SPE/AuNP/Apt/Ara h 1 from AFM images (Fig. 4).

The observed decrease in surface roughness (S_a_) following aptamer immobilization is driven by a molecular levelling mechanism. The electrodeposited AuNPs create a highly nanostructured and porous topography characterized by sharp protrusions and deep interstitial voids (valleys). Upon the self-assembly of the thiolated truncated aptamers, these biomolecules preferentially occupy the micro-cavities and ‘valleys’ of the gold matrix due to their flexible single-stranded nature. This ‘gap-filling’ process masks the initial vertical deviations of the AuNP layer, resulting in a more uniform and planar bio-interface. This structural reorganization leads to a reduction in the S_a_ value from 61.04 nm to 45.81 nm, providing a stable and organized foundation for the subsequent capture of the Ara h 1 target, which then induces a secondary roughening of the surface (127.72 nm).

The gold nanostructures were characterized directly on the electrode surface to maintain their structural integrity as synthesized. Surface-sensitive techniques, including SEM/EDX for elemental and morphological confirmation and AFM for topographical analysis, were prioritized over solution-based methods like UV-Vis or TEM. This approach ensures that the characterized state of the AuNPs accurately represents the platform used for subsequent biosensing applications.

### Analytical performance of the aptasensor

**Fig. 5:**
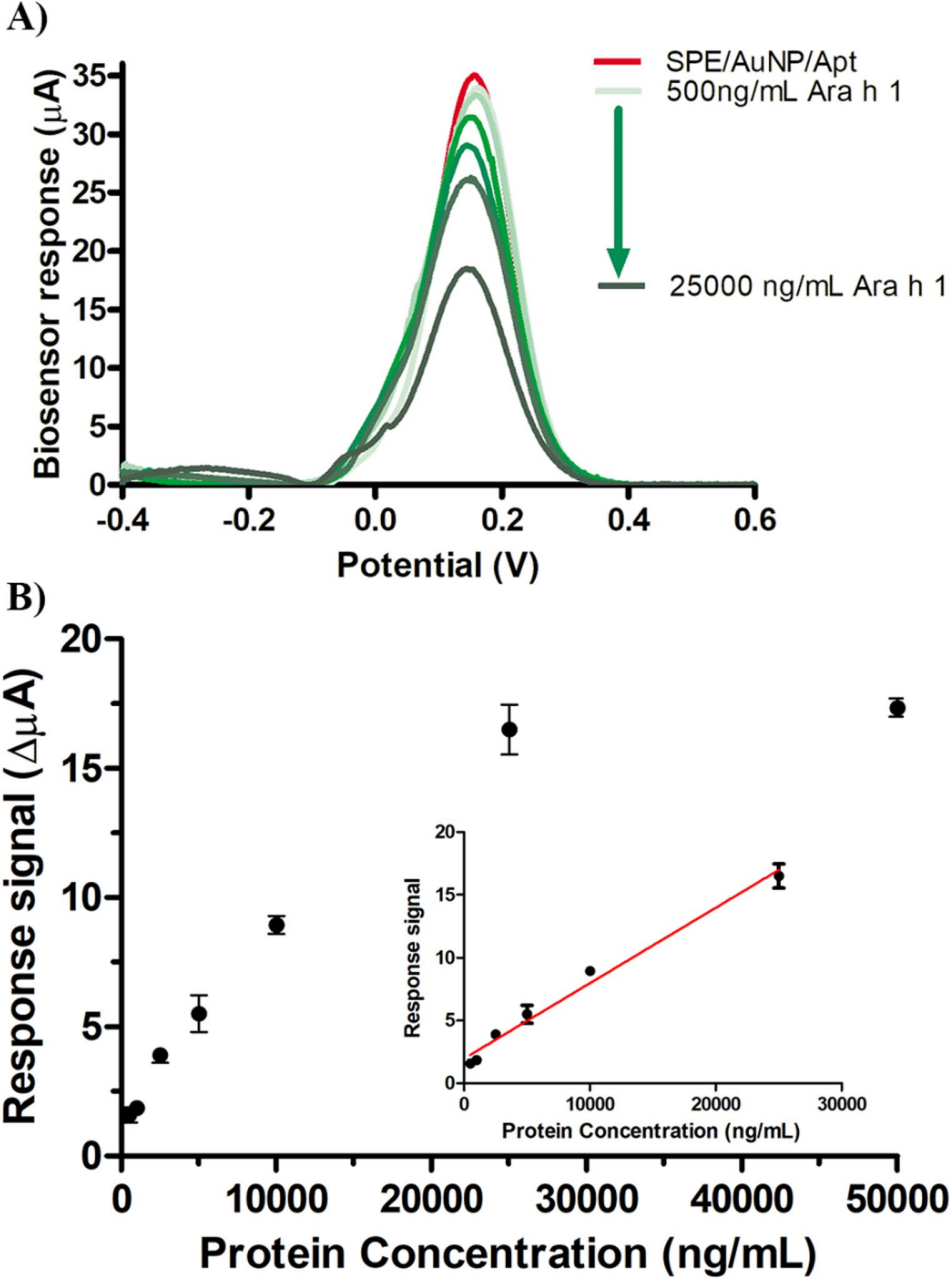
DPV graphs (**A**) and Calibration curve (**B**) of SPE/AuNP/Apt sensor

To evaluate the analytical performance of the developed aptasensor, 5µL of Ara h 1 protein solutions with concentrations ranging from 500 to 50,000 ng/mL were applied to the SPE/AuNP/Apt surface (Fig. 5). Within the 500–25,000 ng/mL concentration range, the sensor’s response showed a direct linear correlation, described by the equation y = 0.0006x + 1.98 (R² = 0.9836). The sensitivity was calculated as 0.0085 ΔµA ng^−1^mL cm^− 2^,using formula slope of the calibration curve/electrode area. The limit of detection (LOD) was calculated as 500 ng/mL using the formula 3Sd/m, where Sd is the standard deviation derived from the lowest concentration of the analyte and m is the slope of the calibration curve. The obtained results were compared with the performance of various biosensors reported in recent years, as summarized in Table 1. The comparison included different biosensors and analytical approaches. The study demonstrated SPE/AuNP/Apt LOD value close to works reported in the Table 1, alongside a significantly broader linear detection range. Considering the analytical characterizations of the SPE/AuNP/Apt, the aptasensor appears to be a promising alternative for Ara h 1 detection.

**Table 1:** Comparison of the performance of different analytical methods for Ara h 1 detection

Detection method	Detection range (ng/mL)	Detection limit (ng/mL)	Detection time (min)	References
Nanobead-enhanced SPR biosensor	10–2000	90	20	[50]
Linear sweep voltammetry	1–1000	1	30	[51]
Cyclic voltammetry-Differential pulse voltammetry	50 −1000	21.6	20	[52]
Fluorescence	0,1–100	0,04	80	[53]
Colorimetric	125–4000	25.0	60	[54]
Lateral flow assay (LFA)	10–5000	10	45	[55]
Enzyme-linked immunosorbent assay (ELISA)	50–20,000	10	90	[56]
Electrochemical immunosensor	20.8–1000	6.3	120	[2]
Electrochemical immunosensor	12.6–2000	3.8	230	[57]
Electrochemical aptasensor	5–150 nM	1.66 nM	90	[58]
Electrochemical aptasensor	Qualitative sensor			[59]
Electrochemical aptasensor	500–25000	500	60	This work

**Fig. 6:**
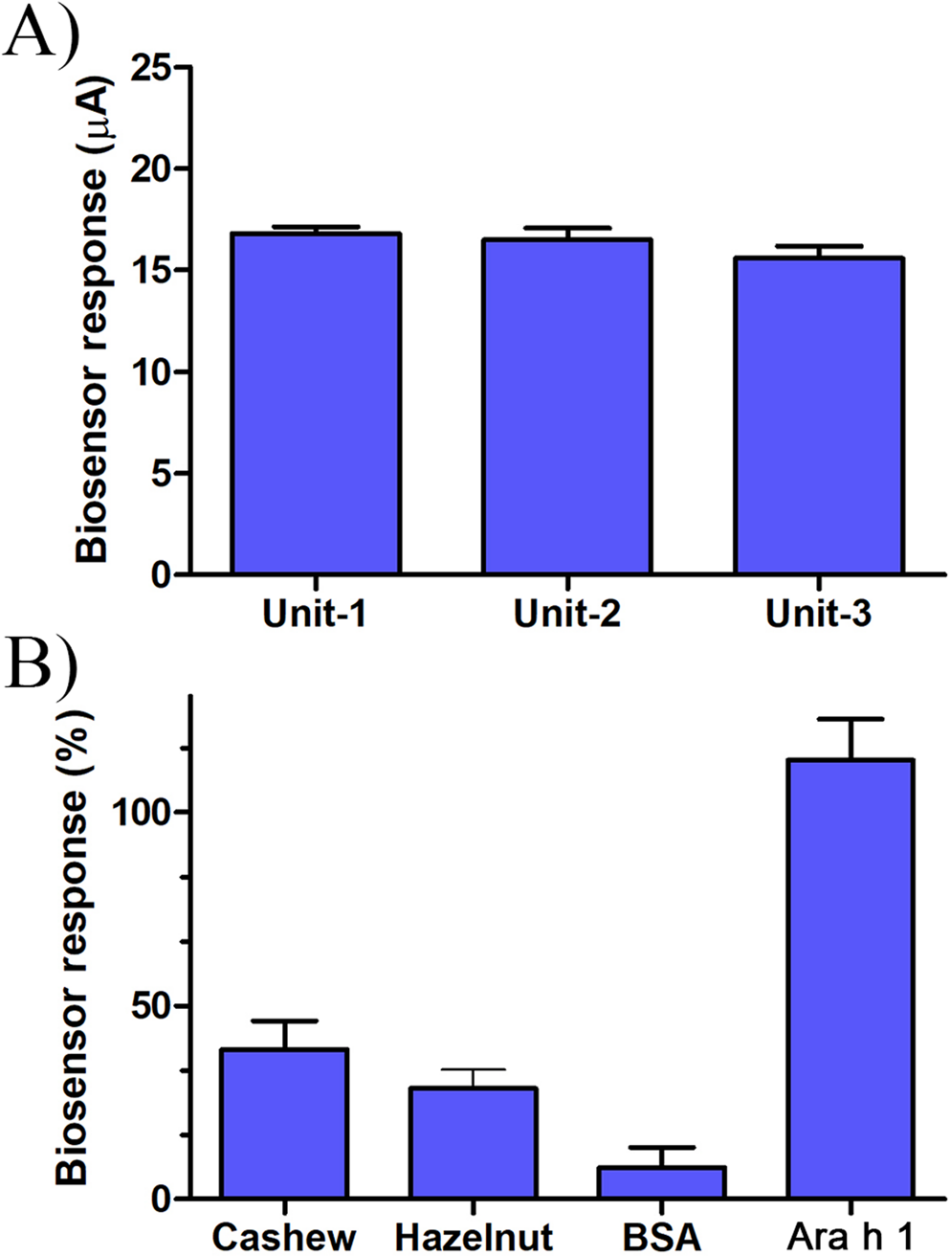
Repeatability and reproducibility of the sensor (**A**) and interferents effect on SPE/AuNP/Apt sensor (**B**)

For reproducibility and repeatability studies, the SPE/AuNP/Apt sensor was prepared using three different electrodes, and 10 measurements were taken for each with a 25,000 ng/mL Ara h 1 concentration (Fig. 6A). The results are presented in Fig. 6a. Additionally, the overall standard deviation and coefficient of variation (%CV) calculated from all data were ± 0.719 and 4.42%, respectively without significant differences between electrodes. The analytical performance of the SPE/AuNP/Apt were summarized in Table S4.

To evaluate the selectivity of the biosensor, potential interferents such as bovine serum albumin (BSA), cashew, and hazelnut allergen proteins were tested at a concentration of 5000 ng/mL using the SPE/AuNP/Apt sensor (Fig. 6B). Despite the molecular structures of cashew and hazelnut allergen proteins being highly similar to that of Ara h 1, the sensor response was only approximately 20–35%. These results indicate that the aptasensor exhibits high selectivity towards Ara h 1.

The stability and shelf-life of the developed SPE/AuNP/Apt electrodes were evaluated based on their operational consistency. While the current protocol focuses on ‘on-site’ fresh modification and immediate analysis to ensure maximum bio-interface integrity, preliminary assessments and literature data on similar thiolated aptamer-gold nanoparticle systems suggest that the electrodes maintain approximately 90% of their initial response when stored at 4 °C under dry conditions for up to 7 days. A comprehensive long-term stability study, including varying storage temperatures and humidity levels, will be the focus of future work to further enhance the commercial viability of this portable platform.

### Real sample analysis

**Fig. 7:**
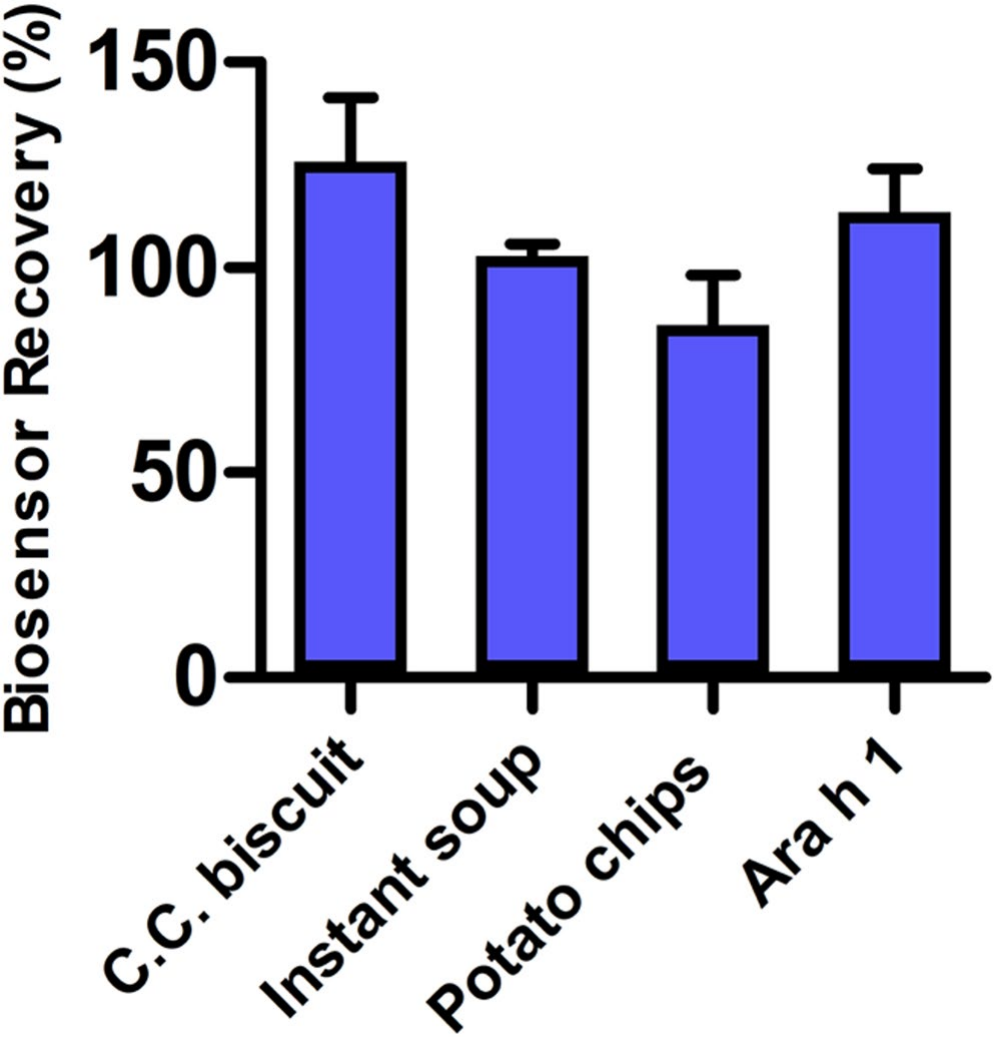
Real samples’ (spiked with 5000 ng/mL Ara h 1) results were obtained with mobile phone potentiostat

The practical applicability of the aptasensor was further validated by spiking complex food matrices with Ara h 1 at a concentration of 5000 ng/mL (5 ppm). As shown in Fig. 7, the smartphone-integrated platform successfully recovered the allergen with high accuracy across all samples. This concentration (5 ppm) was selected as a representative ‘action level’ for industrial cross-contamination monitoring. The ability to detect 5 ppm Ara h 1 in untreated or minimally processed matrices—where standard ELISA might require hours of lab work—highlights the efficiency of our mobile electrochemical platform.

For the analytical validation of the sensor’s accuracy and to eliminate the ‘nugget effect’ commonly associated with heterogeneous allergen distribution in food, the spiking method was employed. This allowed for the precise calculation of recovery rates across different complex matrices. Future studies will focus on the application of the platform to naturally contaminated industrial samples to further evaluate its performance in uncharacterized field conditions.

Real sample testing was conducted using instant soup, potato chips, and caramel-coated chocolate. The preparation method of the samples was described in the experimental section, and 5000 ng/mL Ara h 1 was included into each. Measurements were performed using a mobile potentiostat connected to a smartphone. Based on the results for 5000 ng/mL Ara h 1 prepared in buffer solution and Ara h 1-spiked samples, the biosensor’s % recoveries were calculated and summarized in Table S5. Using a mobile phone, recovery rates of 125.9%, 102.8%, and 86.0% were obtained for the caramel-coated chocolate, instant soup, and potato chip samples, respectively. The overall recovery rate for Ara h 1 detection was 113.4%.

To evaluate the impact of dissolved matrix components on the sensor’s accuracy, the final 10-fold diluted food extracts were spiked with Ara h 1 to reach a concentration of 5000 ng/mL (5 ppm). This approach allowed for a direct assessment of recovery rates in the presence of complex interferences (e.g., lipids and carbohydrates from caramel-coated chocolate) without the confounding variables of extraction efficiency.

While the current protocol utilizes a 250-fold dilution to ensure robust performance across all tested matrices, the platform offers the flexibility to achieve lower matrix-equivalent LODs. By optimizing the sample-to-buffer ratio and reducing the secondary dilution for less complex food types, the system can be calibrated to detect Ara h 1 at concentrations closer to the intrinsic buffer-LOD (0.5 ppm), depending on the specific industrial requirements.

To address the regulatory significance of the developed sensor, its performance was compared with established food safety standards and gold-standard commercial ELISA kits. According to the VITAL 3.0 guidance, the reference dose for peanut protein is 2.0 mg, which translates to action levels in the low ppm range depending on the serving size. Our sensor’s LOD of 0.5 ppm (500 ng/mL) and the successfully demonstrated detection of 5 ppm (5000 ng/mL) in complex matrices (Fig. 7) fall squarely within the relevant industrial monitoring thresholds. As shown in Table 1, while some lab-based sensors offer lower LODs in pure buffers, our platform matches the functional detection limit of commercial ELISA kits (~ 0.5–1.0 ppm) while providing a much faster response time (< 30 min) and the convenience of smartphone integration, making it a viable tool for industrial food safety compliance.

## Conclusion

This study successfully demonstrates a smartphone-integrated electrochemical aptasensor for the rapid and selective detection of the peanut allergen Ara h 1. The novelty of the platform lies in the first-time utilization of a truncated Ara h 1 aptamer sequence, which, when combined with electrodeposited gold nanoparticles, provides a robust and cost-effective bio-interface. Topographical analysis via AFM confirmed a unique ‘gap-filling’ effect, where the truncated aptamers stabilized the surface morphology (reducing roughness to 45.81 nm) before the selective capture of the bulky allergen.

The developed biosensor exhibited a broad linear detection range (500–25,000 ng/mL) and high selectivity against structural homologs such as cashew and hazelnut allergens. When applied to complex food matrices (chocolate, soup, and chips) via a portable potentiostat, the system achieved a successful overall recovery rate of 113.4%, demonstrating its potential for decentralized food safety monitoring.

Despite these advancements, the study has certain limitations. While the detection limit is sufficient for identifying significant cross-contamination events in industrial production lines (approx. 5 ppm in food samples), further signal amplification strategies may be required to meet the most stringent regulatory thresholds for ‘allergen-free’ labeling (0.5–5 ppm). Our study included recovery calculations based on Ara h 1 spiked into the food extract. Therefore, the calculated recovery values reflect the analytical performance of the aptamer-based detection system, rather than the extraction efficiency from the food matrix. The spiked samples were prepared by adding known concentrations of the allergen protein to the food extracts, as described in previous studies [60, 61]. In our study, we spiked the allergen protein after extraction to assess the analytical performance of the aptamer in a food matrix. However, a comprehensive evaluation of the method requires spiking the raw food with the allergen prior to extraction. Additionally, while the sensor shows high operational stability, long-term shelf-life studies under varying environmental conditions are necessary for full commercial deployment. Future research will focus on integrating a multiplexed array to detect multiple allergens simultaneously and enhancing the sensitivity through secondary nanomaterial modifications. Overall, this smartphone-based platform bridges the gap between lab-scale precision and field-ready diagnostics, offering a promising tool for real-time allergen screening.

## Supplementary Information

Below is the link to the electronic supplementary material.

Supplementary Material 1 (DOCX 1.12 MB)

Supplementary Material 2 (JPG 2.05 MB)

Supplementary Material 3 (JPG 2.42 MB)

Supplementary Material 4 (DOCX 1.12 MB)

Supplementary Material 5 (DOCX 1.25 MB)

Supplementary Material 6 (DOCX 1.25 MB)

## Data Availability

No datasets were generated or analysed during the current study.
