# Supplementary material for: A truncated aptamer-based electrochemical sensor for sensitive Ara h 1 determination on gold nanoparticle-modified screen-printed electrodes
Source: Mikrochim Acta. 2026 Jun 5;193(7):444. doi: 10.1007/s00604-026-08153-w (PMC13236829; doi:10.1007/s00604-026-08153-w)
Supplement: Supplementary file 1 — Supplementary Material 1 (DOCX 1.12 MB) [file 604_2026_8153_MOESM1_ESM.docx]

**Supporting Information**

**Gold Nanoparticle Decorated Screen Printed Electrode Based Mobile Electrochemical Aptasensor for Detection of Peanut Allergen Ara h 1**

Serdar ŞANLI ^a^, Songül Kırlak Kara ^a^, Burhan Bora ^b^, Serkan Şen ^a^, Mutlu Sönmez Çelebi ^a^, Serap Evran ^b^.

^a^ Department of Chemistry, Faculty of Science and Arts, Ordu University, 52200, Altınordu, Ordu, Türkiye

^b^ Department of Biochemistry, Faculty of Science, Ege University, Bornova, Izmir 35100, Türkiye

**Optimization of food extraction buffer**

In order to increase the recovery of aptasensor, food extraction buffer was optimized. Regarding the binding behavior of aptamer to its target protein PBS and Tris buffers were chosen to test their efficiency. Protein extraction buffer that is being supplied by peanut ELISA kit by manufacturer (Rida) was also used to compare the efficiency of in house optimized buffers. 50 mM Tris-HCl pH:8.0 including 150 mM NaCl, PBS pH:7.4 supplemented with 2 mM MgCl_2_ were prepared before use. Food samples obtained from local market grinded or finely cut depending on the sample type, and 1 gram of sample was added on 20 mL of extraction buffer to be tested. Samples were shaken in and incubator set to 60 ^o^C for 30 minutes. After centrifugation at 21.000 g for 30 minutes, soluble protein containing supernatant was transferred to a new tube, avoiding to take any fat layer. Protein concentration was determined according to Bradford protein assay, using BSA as a standard. Results are given at table S1 and figure S1. Regarding the target binding ability of Ara h 1 aptamer, PBS-T buffer was chosen for protein extraction from food samples. Same buffer was used as a binding buffer for developed aptasensor. Since the extraction buffer and working buffer is the same, there is no need for protein dilution after extraction procedure and this advantage serves to increase the sensitivity of our aptasensor.

**Table S1.** Comparison of protein extraction buffers. Tris; Tris-T1 and Tris-T5: 0, 0,1 and 0,5% tween-20 supplemented 50 mM Tris-HCl pH:8.0 respectively; PBS, PBS-T1 and PBS-T5: 0, 0.1 and 0.5 Tween-20 supplemented PBS respectively; Rida: commercial Ridascreen peanut allergen kit buffer. Protein concentration was determined according to Bradford protein assay.

| **Buffer** | **Tris** | **Tris-T1** | **Tris-T5** | **PBS** | **PBS-T1** | **PBST5** | **Rida** |
| --- | --- | --- | --- | --- | --- | --- | --- |
| **Protein (µg/mL)** | 329.56±15 | 337.27±9.4 | 370.42±33 | 273.37±10.4 | 297.45±19.7 | 298.35±4.8 | 324.46±6.7 |
| **%protein** | 88.99±3.6 | 91.28±6.5 | 99.65±0.5 | 73.86±3.6 | 80.18±1.8 | 80.80±6.2 | 88.09±9.1 |


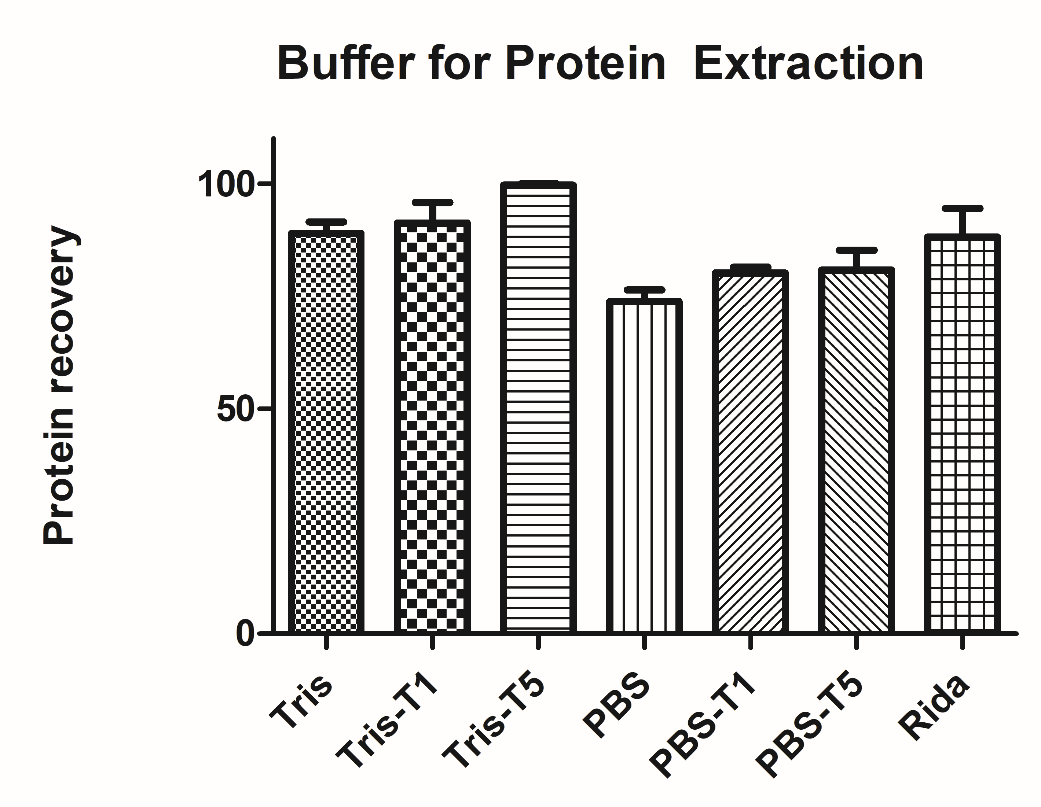


**Figure S1.** Comparison of protein extraction buffers. Tris; Tris-T1 and Tris-T5: 0, 0,1 and 0,5% tween-20 supplemented 50 mM Tris-HCl pH:8.0 respectively; PBS, PBS-T1 and PBS-T5: 0, 0.1 and 0.5 Tween-20 supplemented PBS respectively; Rida: commercial Ridascreen peanut allergen kit buffer. Protein concentration was determined according to Bradford protein assay.

Amino acid sequences of allergen proteins used in this study given at figure S2. Genes encoding allergen proteins were cloned to pET-28a expression vector and protein expression was done with E. coli T7 Express Iq. For the prooagation of heterologous protein expression 10 mL of overnight culture was inoculated to 1 L sterile LB medium and incubated at 37 ^o^C until OD_600_ reached to 0.6. At this point, growth media was supplemented with 0.3 mM IPTG and incubation temperature was set to 18 ^o^C. After incubating bacterial culture for 36 hours, cells were harvested by centrifugation and bacterial pellet was resuspended in lysis buffer (100 mM KH_2_PO_4_ pH:7.8, 500 mM NaCl, 20 mM imidazole, %0.1 Triton X-100). Ultrasonification was carried out on ice bath using vibracell ultrasonic processor. Crude lysate centrifuged at 21.000 g and clear supernatant with soluble protein fraction was syringe-filtered through 0.45 µm filter. Filtrate was loaded to Cytiva HisTrap FF Crude column connected to AKTA FPLC system and the column eas extensively washed (10xCV) with wash buffer after injection in order to get rid of non-specific binders (Wash buffer: lysis buffer without Triton X-100). Gradient elution was started by increasing percentage of elution buffer gradually (elution buffer: 500 mM imidazole in wash buffer). Elution of the protein was monitored according to UV absorbance of protein at 280 nm wavelength. Fractions collected and purest fractions were combined according to SDS-PAGE analysis. Gradient elution chromatogram of allergen proteins is given in figure S3. Chromatograms evidence for good separation of recombinant proteins from impurities as it was also evidenced by SDS-PAGE analysis given in figure S4.


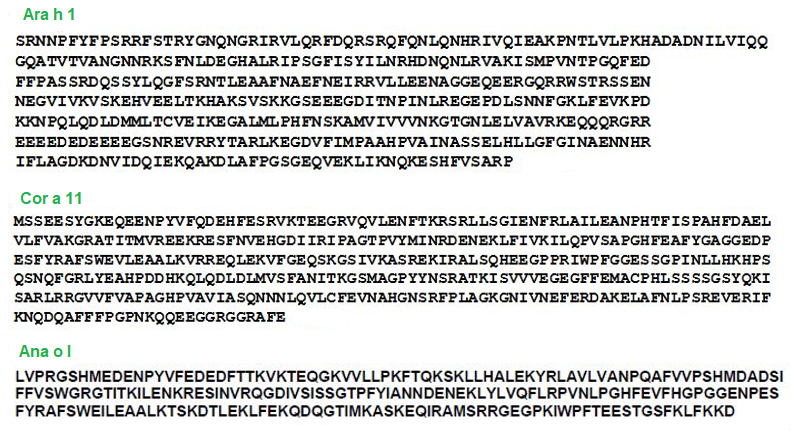


**Figure S2.** Amino acid sequences of recombinant allergen proteins used in this study.


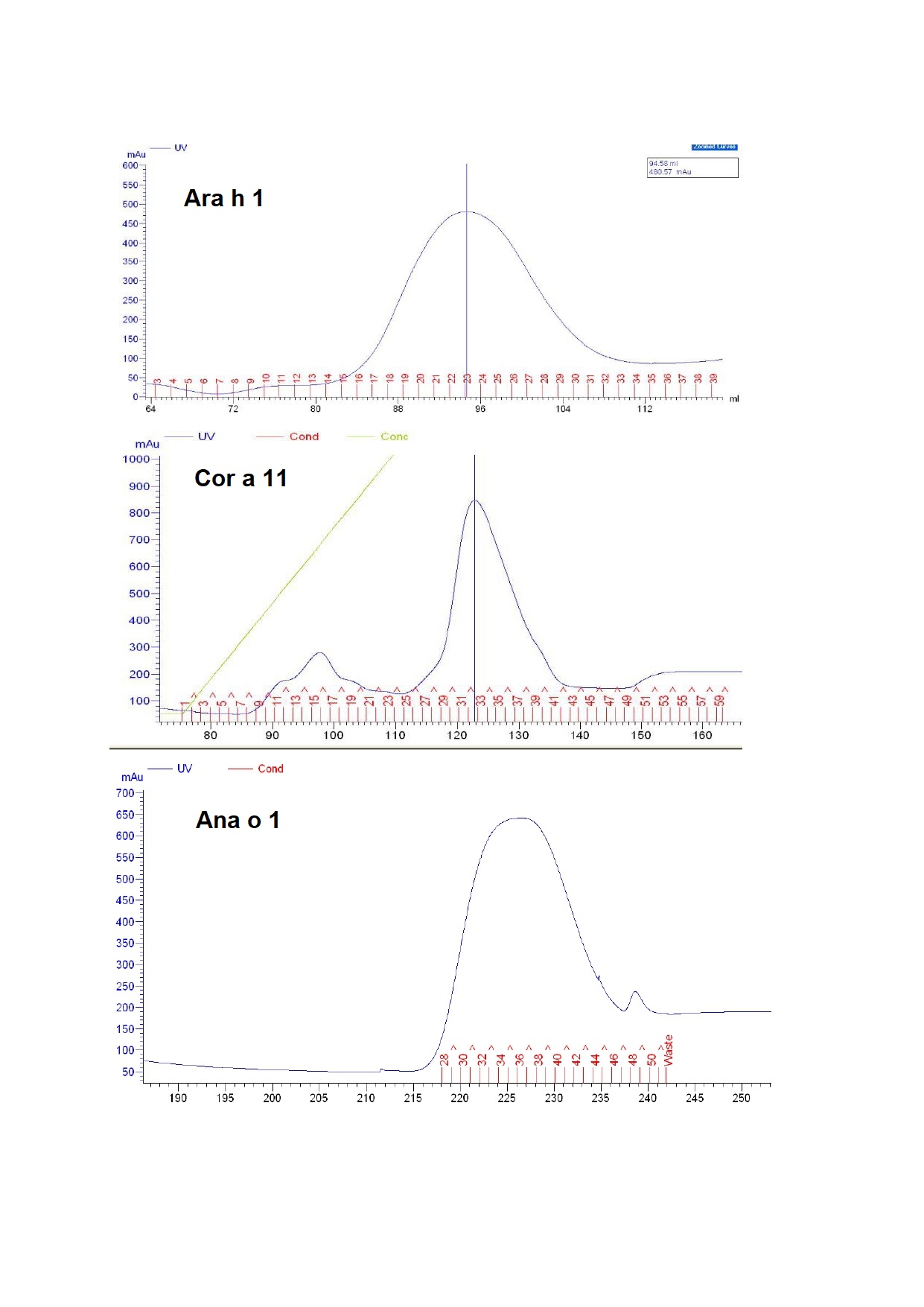


**Figure S3.** Gradient elution chromatogram of allergen proteins.


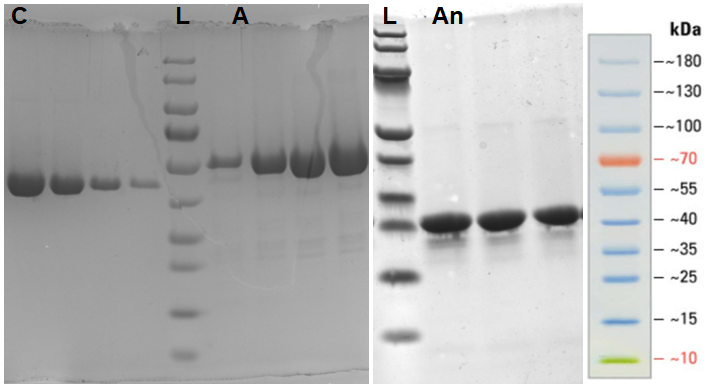


**Figure S4.** SDS PAGE analysis of recombinant Ara h1, Cor a 11 and Ana o I. C: Cor a 11; A: Ara h 1; An: Ana o I; L: PageRuler prestained protein ladder.

In order to check reactivity of recombinant allergen protein Ara h 1 we utilized R&D Systems Ridascreen peanut allergen ELISA kit. We repeated the procedure given in the manual of the kit. Protein samples were diluted in the protein extraction buffer provided in the kit. All samples and standards were incubated in the antibody-immobilized wells for 1 hour. After incubation wells were washed with the wash buffer supplied in the kit. HRP-conjugated secondary antibody solution was transferred to the wells and incubated for another 1 hour. After the wash step, color reagent was transferred to the wells and the color reaction was incubated in the dark. After the development of blue color 2 M sulfuric acid solution was added to the wells in order to stop color reaction and stabilize the oxidized TMB. ELISA test results given in the figure S5. According to the ELISA test, recombinant allergen Ara h 1 is reactive to antibodies provided by R&D Systems.


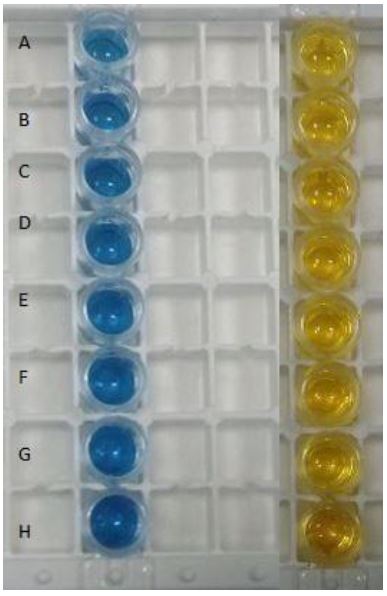


**Figure S5.** ELISA reactivity of recombinant Ara h 1 protein. A: standard protein extract; B, C and D: Protein expression cell lysate; E and F: purified recombinant Ara h 1; G and H: serially diluted recombinant Ara h 1. R&D Biosystems peanut allergen kit.

**Table S2.** Cyclic voltammetry characterization results.

|  | anodic peaks | cathodic peaks | peak to peak separation |
| --- | --- | --- | --- |
| SPE | 40,756 | -40,546 | 0,22 V |
| SPE/AuNP | 62,41 | -62,291 | 0,120 V |
| SPE/AuNP/Apt | 60,809 | -58,132 | 0,140 V |
| SPE/AuNP/Apt/ Ara h 1 | 49,107 | -46,383 | 0,180 V |

**Table S3.** The analytical performance of the SPE/AuNP/Apt

| LOD | 500 ng/mL |
| --- | --- |
| Lineer range | 500-25000 ng/mL |
| Detection time | 60 min |
| % CV | ± 0,719 %4,42 |

**Table S4.** Biosensor % recoveries for real samples.

|  | % recoveries | standart devision |
| --- | --- | --- |
| C. c. biscuit | 125,92 | 15,47 |
| Instant soup | 102,81 | 2,93 |
| Potato chips | 86,06 | 12,13 |
| Ara h 1 | 113,46 | 10,65 |
