# Supplementary figures and images for: A truncated aptamer-based electrochemical sensor for sensitive Ara h 1 determination on gold nanoparticle-modified screen-printed electrodes
Source: Mikrochim Acta. 2026 Jun 5;193(7):444. doi: 10.1007/s00604-026-08153-w (PMC13236829; doi:10.1007/s00604-026-08153-w)

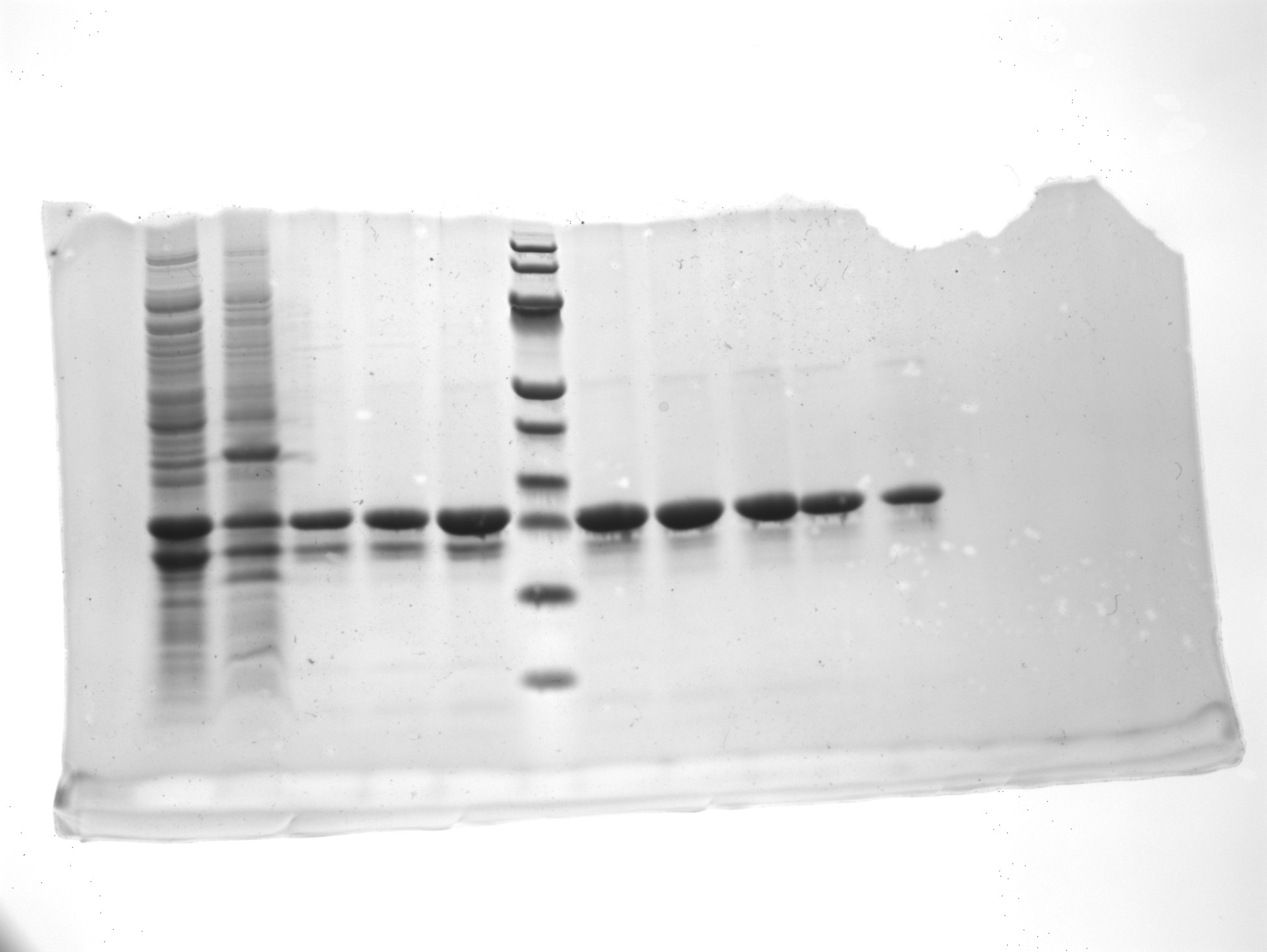

Supplement: Supplementary file 2 — Supplementary Material 2 (JPG 2.05 MB) [file 604_2026_8153_MOESM2_ESM.jpg]

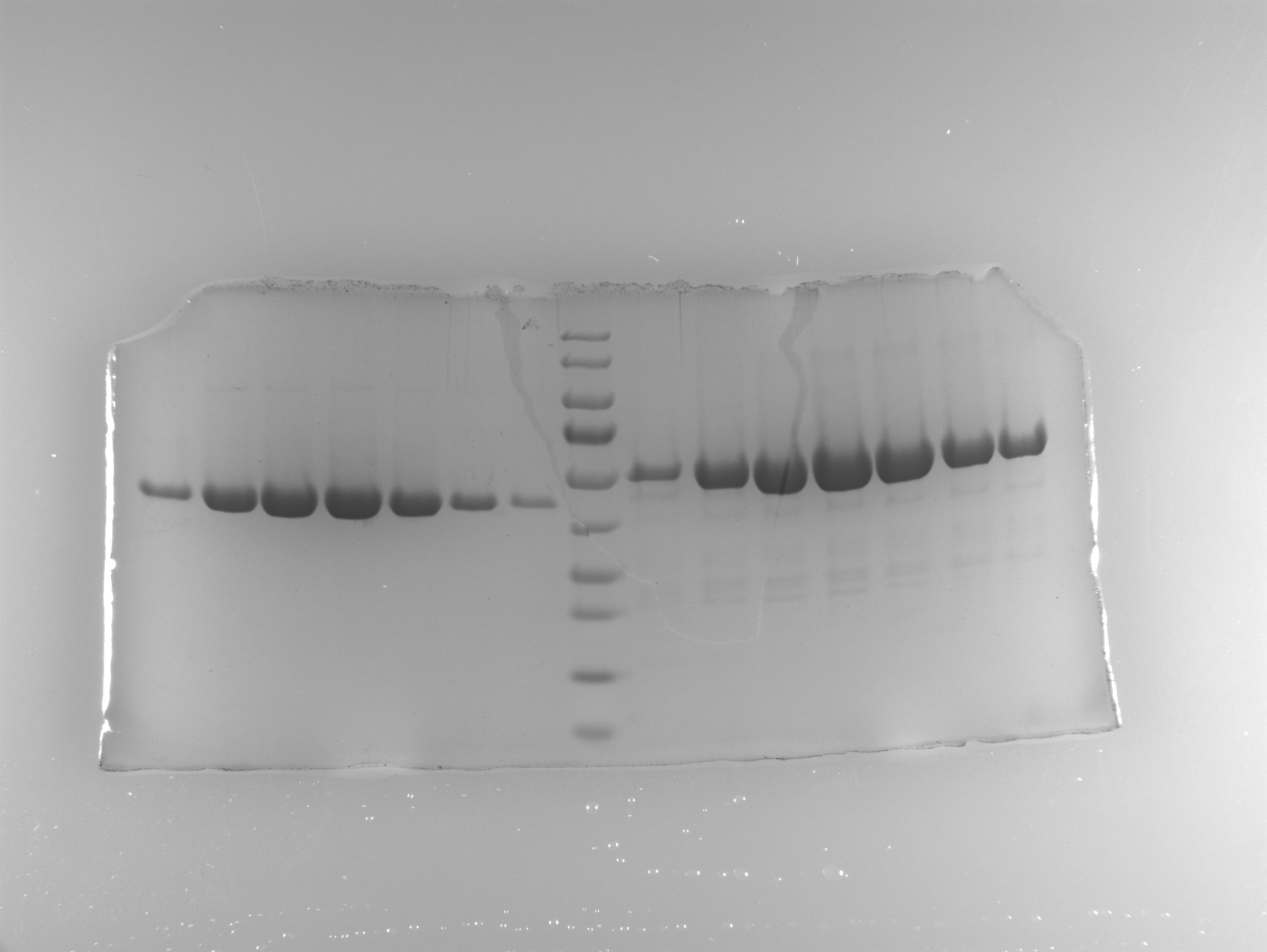

Supplement: Supplementary file 3 — Supplementary Material 3 (JPG 2.42 MB) [file 604_2026_8153_MOESM3_ESM.jpg]
